# Emergence of Dynamically‐Disordered Phases During Fast Oxygen Deintercalation Reaction of Layered Perovskite

**DOI:** 10.1002/advs.202301876

**Published:** 2023-04-25

**Authors:** Takafumi Yamamoto, Shogo Kawaguchi, Taiki Kosuge, Akira Sugai, Naoki Tsunoda, Yu Kumagai, Kosuke Beppu, Takuya Ohmi, Teppei Nagase, Kotaro Higashi, Kazuo Kato, Kiyofumi Nitta, Tomoya Uruga, Seiji Yamazoe, Fumiyasu Oba, Tsunehiro Tanaka, Masaki Azuma, Saburo Hosokawa

**Affiliations:** ^1^ Laboratory for Materials and Structures Institute of Innovative Research Tokyo Institute of Technology Yokohama 2268503 Japan; ^2^ Japan Synchrotron Radiation Research Institute (JASRI) SPring‐8, 1‐1‐1 Kouto Sayo‐gun Hyogo 6795198 Japan; ^3^ Institute for Materials Research Tohoku University 2‐1‐1 Katahira, Aoba‐ku Sendai 9808577 Japan; ^4^ Department of Applied Chemistry for Environment Graduate School of Urban Environmental Sciences Tokyo Metropolitan University 1‐1 Minami‐Osawa Hachioji Tokyo 1920397 Japan; ^5^ Department of Chemistry Graduate School of Science Tokyo Metropolitan University 1‐1 Minami‐Osawa Hachioji Tokyo 1920397 Japan; ^6^ Elements Strategy Initiative for Catalysts & Batteries (ESICB) Kyoto University Katsura, Nishikyo‐ku Kyoto 6158245 Japan; ^7^ Department of Molecular Engineering Graduate school of Engineering Kyoto University Nishikyo‐ku Kyoto 6158510 Japan; ^8^ Living Systems Materialogy (LiSM) Research Group International Research Frontiers Initiative (IRFI) Tokyo Institute of Technology Yokohama 2268501 Japan; ^9^ Kanagawa Institute of Industrial Science and Technology (KISTEC) 705‐1 Shimoimaizumi Ebina Kanagawa 2430435 Japan; ^10^ Faculty of Materials Science and Engineering Kyoto Institute of Technology Matsugasaki Sakyo‐ku Kyoto 6068585 Japan

**Keywords:** oxygen storage material, perovskite oxide, synchrotron X‐ray technique, time‐resolved measurement, topochemical reaction

## Abstract

Determination of a reaction pathway is an important issue for the optimization of reactions. However, reactions in solid‐state compounds have remained poorly understood because of their complexity and technical limitations. Here, using state‐of‐the‐art high‐speed time‐resolved synchrotron X‐ray techniques, the topochemical solid‐gas reduction mechanisms in layered perovskite Sr_3_Fe_2_O_7−__*δ*_ (from *δ* ∼ 0.4 to *δ* = 1.0), which is promising for an environmental catalyst material is revealed. Pristine Sr_3_Fe_2_O_7−__*δ*_ shows a gradual single‐phase structural evolution during reduction, indicating that the reaction continuously proceeds through thermodynamically stable phases. In contrast, a nonequilibrium dynamically‐disordered phase emerges a few seconds before a first‐order transition during the reduction of a Pd‐loaded sample. This drastic change in the reaction pathway can be explained by a change in the rate‐determining step. The synchrotron X‐ray technique can be applied to various solid‐gas reactions and provides an opportunity for gaining a better understanding and optimizing reactions in solid‐state compounds.

## Graphical abstract

The topochemical solid‐gas reduction mechanisms from Sr_3_Fe_2_O_7−__*δ*_ to Sr_3_Fe_2_O_6_ is investigated by high‐speed time‐resolved synchrotron X‐ray techniques. The reaction pathways drastically change by Pd‐loading, and a dynamically‐disordered phase emerges a few seconds during the reduction of the Pd‐loaded Sr_3_Fe_2_O_7−__*δ*_. This synchrotron X‐ray technique will provide an opportunity for gaining a better understanding and optimizing various solid‐gas reactions.

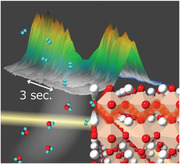

## 1 Introduction

Understanding the mechanisms behind chemical reactions is one of the biggest issues in materials chemistry, since this makes it possible to create compounds with the desired structures or to optimize reactions toward functional uses. However, reactions in solid‐state crystalline compounds are rather poorly understood^[^ ^1^ ^]^ compared with molecular reactions whose steps have been analyzed with nuclear magnetic resonance (NMR) and other spectroscopies. This is partly because solid‐state reactions generally require high temperatures; thus, it is relatively difficult to monitor them by using in‐situ methods. Inhomogeneous reactions derived from the difference in surface and bulk states as well as the existence of numerous numbers of atoms in crystals also prevent precise analyses of reactions in solid states. In order to achieve the rational design of solid‐state compounds and their reactions, the development of in‐situ measurement techniques is necessary.

In‐situ X‐ray diffraction (XRD) is a powerful tool for monitoring reactions in bulk materials,^[^ ^2^ ^]^ and the recent development of synchrotron X‐rays enables us to access high‐resolution data with a short time window. Time‐resolved synchrotron XRD measurements have been used to investigate many reactions in crystalline phases, such as solid‐state reactions,^[^ ^26^ ^]^ hydrothermal reactions,^[^ ^3^ ^]^ gas absorption,^[^ ^4^, ^5^ ^]^ and solid‐gas catalytic reactions.^[^ ^6^ ^]^ However, the development of a time‐resolved XRD measurement of a solid‐gas reaction that can reveal structural evolution precisely on a subsecond scale is still a challenge. When the time scale of the measurement becomes faster, one can capture intermediate phases with a shorter lifetime. This will provide further opportunities to develop optimization of the reactions and/or synthesis of metastable structures.

Here, we focus on the Ruddlesden‐Popper layered perovskite Sr_3_Fe_2_O_7−__*δ*_, which has recently attracted attention as a high‐performance oxygen storage material.^[^ ^7^, ^8^, ^9^ ^]^ This compound shows a reversible topochemical redox reaction between Sr_3_Fe_2_O_7−__*δ*_ (*δ* ∼ 0.4) and Sr_3_Fe_2_O_6_ (*δ* = 1.0) under O_2_ and H_2_ at 773 K and excellent performance as an environmental catalyst material. Our previous study revealed that Pd loading dramatically promotes the oxygen release rate and decreases the release temperature under H_2_ flow on Sr_3_Fe_2_O_7−__*δ*_ (*δ* ∼ 0.4),^[^ ^8^ ^]^ but the reaction pathways and structural evolution during the reduction were still unclear. In the study reported here, we monitored the reduction reaction from Sr_3_Fe_2_O_7−__*δ*_ (*δ* ∼ 0.4) to Sr_3_Fe_2_O_6_ by using a high‐speed time‐resolved synchrotron X‐ray diffraction (XRD) technique with the time window of a few hundred milliseconds. We found a gradual single‐phase structural evolution during the reduction of pristine Sr_3_Fe_2_O_7−__*δ*_ (*δ* ∼ 0.4), while 1.0 wt% Pd loading drastically altered the reaction pathway, where there was a first‐order transition from a dynamically‐disordered phase to a stable‐ordered phase. This reaction was further investigated by using the simultaneous quick X‐ray absorption fine structure (QXAFS)–XRD technique. These high‐speed synchrotron X‐ray techniques allow us to determine the reaction pathways of the fast solid‐gas reactions and will provide an opportunity to understand and optimize reactions in solid‐state compounds.

## 2 Results and Discussion

### 2.1 High‐Speed Time‐Resolved XRD Measurements for the Topochemical Reductions of Sr_3_Fe_2_O_7−__*δ*_ and Pd/Sr_3_Fe_2_O_7−__*δ*_

**Figure 1:**
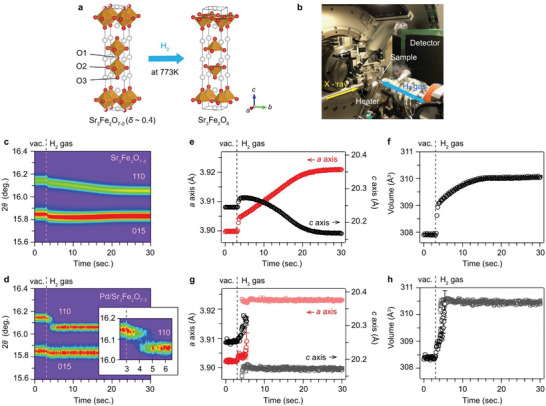
High‐speed time‐resolved XRD measurements for the reduction reaction of Sr_3_Fe_2_O_7−__*δ*_ at 773 K. a) Reduction reaction of Sr_3_Fe_2_O_7−__*δ*_ by H_2_ gas. The crystal structures are visualized by VESTA.^[^ ^10^ ^]^ b) Experimental setup around the sample. c) Time‐resolved XRD patterns for the reduction of Sr_3_Fe_2_O_7−__*δ*_ and d) Pd/Sr_3_Fe_2_O_7−__*δ*_. The inset in **d** is a magnification of the 110 peak around the location of H_2_ gas injection. Time profiles of the lattice parameters for e,f) Sr_3_Fe_2_O_7−__*δ*_ and g,h) Pd/Sr_3_Fe_2_O_7−__*δ*_.

A pristine Sr_3_Fe_2_O_7−__*δ*_ and 1.0 wt% Pd‐loaded sample (Pd/Sr_3_Fe_2_O_7−__*δ*_) were prepared as reported previously.^[^ ^8^, ^11^ ^]^ The XRD patterns for Sr_3_Fe_2_O_7−__*δ*_ did not change by Pd‐loading (Figure S1, Supporting Information) and the peaks corresponding to Pd species were not detected due to the small amount of Pd. In a static XAFS measurement of Pd‐loaded sample before reduction, the Pd K‐edge extended X‐ray absorption fine structure (EXAFS) oscillation in Pd/Sr_3_Fe_2_O_7−__*δ*_ was different from that of PdO as a standard sample and resembled with Fe K‐edge EXAFS oscillation in Pd/Sr_3_Fe_2_O_7−__*δ*_ (Figure S2, Supporting Information). These results imply that most Pd species were substituted in the Fe site of Sr_3_Fe_2_O_7−__*δ*_, rather than existing as PdO. Thermogravimetric analyses confirmed that the oxygen deficiency *δ* under O_2_ at 773 K was ≈0.4, regardless of the presence of Pd (Figure S3, Supporting Information). In the stable structure of Sr_3_Fe_2_O_7−__*δ*_ (*δ* ∼ 0.4), most of the vacancies are at the O1 site in the perovskite layer, which consists of the apical sites between FeO_6_ octahedra (**Figure** **1**a).^[^ ^12^ ^]^

High‐resolution time‐resolved XRD measurements were carried out on the BL02B2 beamline at SPring‐8 (Figure 1b). The data collection for the reduction was started under vacuum conditions after a pretreatment at 973 K (details are in the experimental section). The interval between the diffraction measurements on Sr_3_Fe_2_O_7−__*δ*_ (or Pd/Sr_3_Fe_2_O_7−__*δ*_) was 200 (or 100) ms. H_2_ gas (≈0.5 atm) was injected 3 seconds after starting the data collection. By injecting H_2_ gas above 773 K, the following reaction proceeds:

(1) $$Sr_{3}Fe_{2}O_{7-\delta }+(1-\delta )H_{2}\to Sr_{3}Fe_{2}O_{6}+(1-\delta )\;\;H_{2}O$$

Regarding the state of Pd species in Pd‐loaded sample, EXAFS oscillations clearly indicate that Pd^2+^ species in Pd‐loaded sample was reduced to Pd metal nanoparticle by the H_2_ reduction at 773 K (Figure S2, Supporting Information). We previously confirmed that the particle size of Pd metal nanoparticle was below 1 nm by comparing the theoretical average coordination number (CN) of mono‐dispersed spherical particles having a cuboctahedral structure with the CN estimated by EXAFS curve fitting analysis.^[^ ^8^ ^]^

Figures 1c,d show the time‐resolved XRD patterns for the reduction of Sr_3_Fe_2_O_7−__*δ*_ and Pd/Sr_3_Fe_2_O_7−__*δ*_ at 773 K (the full angle data are shown in Figures S4 and S5, Supporting Information). We noticed that the sample temperature immediately increased (within 100 ms) when gas was injected. This is because the sample temperature under the vacuum conditions was unintentionally lower than the setting temperature due to cooling by adiabatic expansion. As a result, the peak positions jump to a lower angle immediately after the H_2_ injection (Figure 1c,d). This phenomenon was confirmed by injecting inert gas to Sr_3_Fe_2_O_6_ and Si standard (Figures S6 and S7, Supporting Information). Thermal equilibrium was achieved within 100 ms, and the increase in temperature due to gas injection was estimated to be ≈60 K at 723 K.

It is obvious that the reaction is accelerated by Pd loading. The reaction finishes in ≈30 s for pristine Sr_3_Fe_2_O_7−__*δ*_, while it finishes within a few seconds for Pd/Sr_3_Fe_2_O_7−__*δ*_. This result can be explained by H_2_ spillover effect at the surface.^[^ ^13^, ^14^ ^]^ Most surprisingly, the reaction pathway dramatically changes through promotion of the surface reaction. In the pristine Sr_3_Fe_2_O_7−__*δ*_, the diffraction peaks smoothly move without jumping or broadening (Figure 1c), meaning a single‐phase process (or a second‐order‐like behavior) occurs. In contrast, the 110 peak undergoes an obvious discontinuous jump at ≈4 – 5 s in Pd/Sr_3_Fe_2_O_7−__*δ*_ (inset of Figure 1d), suggesting the existence of a first‐order transition. The inset of Figure 1d and Figure S8, Supporting Information, show the two phases coexisting at 4 – 5 s. The change in the reaction pathway can be also confirmed by examining the time dependence of the lattice parameters obtained by Le Bail analysis (Figure 1e–h). Both the *a*‐ and *c*‐axes of the pristine Sr_3_Fe_2_O_7−__*δ*_ change continuously (Figure 1e), while distinct jumps occur in the lattice parameters of Pd/Sr_3_Fe_2_O_7−__*δ*_ (Figure 1g). Here, we would like to point out that the selectivity of the reaction pathways can be explained by a change of the rate‐determining step, and a nonequilibrium phase appears in the reduction of Pd/Sr_3_Fe_2_O_7−__*δ*_ through fast deintercalation of oxide ions, as will be discussed later.

### 2.2 Simultaneous High‐Speed Time‐Resolved QXAFS – XRD Measurements for the Topochemical Reduction of Pd/Sr_3_Fe_2_O_7−__*δ*_

**Figure 2:**
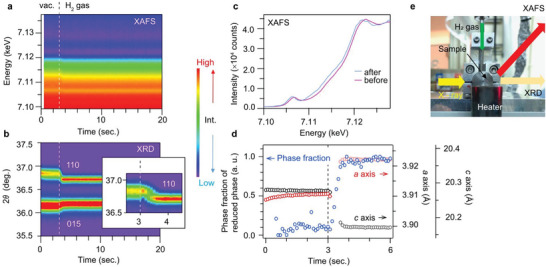
Simultaneous high‐speed time‐resolved QXAFS – XRD measurements for the reduction reaction of Pd/Sr_3_Fe_2_O_7−__*δ*_ at 773 K. a) Time‐resolved Fe K‐edge XANES spectra measured in fluorescence mode. b) Time‐resolved XRD patterns. The inset is a magnification of the 110 peak around the location of the H_2_ gas injection. c) Fe K‐edge XANES spectra of Pd/Sr_3_Fe_2_O_7−__*δ*_ (before) and Pd/Sr_3_Fe_2_O_6_ (after). d) Time profiles of the phase fraction of the final state (blue circles) calculated from fitting using the initial and final states and the lattice parameters (red circles: *a*‐axis, black circles: *c*‐axis). e) Experimental setup around the sample.

We also carried out simultaneous QXAFS and XRD measurements on the BL36XU beamline at SPring‐8 (**Figure** **2**e), to reveal the correlation between the dynamical changes in the electronic and crystal structures. Figure 2a,b show time‐resolved Fe K‐edge X‐ray absorption near edge structure (XANES) spectra and XRD patterns for the reduction reaction of Pd/Sr_3_Fe_2_O_7−__*δ*_ at 773 K. The data were recorded at a 100 ms interval (80 ms and 20 ms exposure times for XAFS and XRD, respectively). The initial condition was a vacuum; then, H_2_ gas (≈98 kPa) was injected 3 s after starting the data collection. The detailed conditions are given in the experimental section.

Both the XANES spectra and the XRD patterns immediately changed after the H_2_ injection (Figure 2a,b). Their reactions finished in 1 s. This is slightly faster than what the data obtained on the BL02B2 beamline show (Figure 1d), probably because of the different experimental condition such as gas pressure. The XANES spectra (Figure 2c) shift to a lower energy state because of the reduction; this finding is consistent with our previous study.^[^ ^9^ ^]^ The jump in the 110 peak at ≈3.3 seconds in the XRD patterns (Figure 2b) can also be seen in Figure 1d. Thus, this result reproduced the reaction observed on BL02B2 (Figure 1d). Figure 2d shows the time dependence of the lattice parameters determined from the XRD data and the fraction of the final state calculated from the fitting using the initial and final states of XANES data. The lattice parameters between 3.2 and 3.5 s are not plotted in Figure 2d due to the low resolution of the data, while two phases coexisting between 3.2 and 3.5 s were clearly seen (Figure S9, Supporting Information). Note that we analyzed the energy shift from the normalized XANES spectra at half of the intensity (Figure S10, Supporting Information), and these results are consistent with the phase fraction in Figure 2d. Remarkably, the phase fraction and the lattice parameter simultaneously change without a time lag, suggesting that the electronic and crystal structures change simultaneously at this time scale.

**Figure 3:**
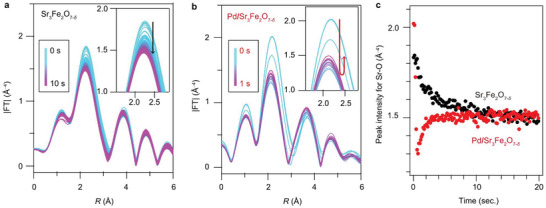
High‐speed time‐resolved DXAFS measurements for the reduction reactions of Sr_3_Fe_2_O_7−__*δ*_ and Pd/Sr_3_Fe_2_O_7−__*δ*_ at 823 K.^[^ ^8^ ^]^ a) Fourier transformations of Sr K‐edge EXAFS spectra for Sr_3_Fe_2_O_7−__*δ*_, and b) Pd/Sr_3_Fe_2_O_7−__*δ*_. H_2_ gas is injected at 0 s. The insets show a magnification of the peak ≈2.2 Å for Sr‐O bonds. c) Time profile of peak intensities for Sr–O bonds ≈2.2 Å.

Previously, we reported nonmonotonic time‐dependence of the Sr K‐edge XANES spectra acquired from energy‐dispersive XAFS (DXAFS) measurements for the reduction of Pd/Sr_3_Fe_2_O_7−__*δ*_.^[^ ^8^ ^]^ On the basis of the suggested findings of the current in‐situ XRD measurements, the Fourier transformed EXAFS spectra acquired after the H_2_ injection were investigated in detail. As shown in **Figure** **3**, the peak at 2.2 Å corresponding to Sr–O bonds in pristine Sr_3_Fe_2_O_7−__*δ*_ gradually decreases, implying that the coordination number of the Sr–O bond decreases through oxygen deintercalation. In contrast, in the case of Pd/Sr_3_Fe_2_O_7−__*δ*_, the peak intensity drops as soon as the H_2_ gas is injected, and it is lower than that of Sr_3_Fe_2_O_6_ between 0.2 and 0.6 s (Figure 3c). This also confirms that Pd loading caused the change in the reaction pathways and the emergence of an intermediate phase.

### 2.3 The Reaction Mechanisms of Sr_3_Fe_2_O_7−__*δ*_ and Pd/Sr_3_Fe_2_O_7−__*δ*_

**Figure 4:**
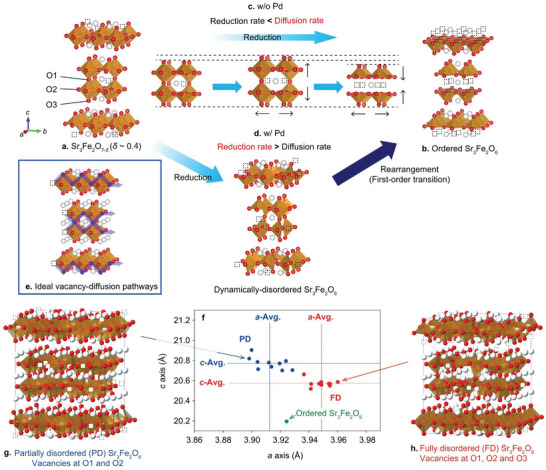
Structural evolution during reduction of Sr_3_Fe_2_O_7−__*δ*_ and Pd/Sr_3_Fe_2_O_7−__*δ*_. a) Schematic structure of Sr_3_Fe_2_O_7−__*δ*_ (*δ* ∼ 0.4). Dotted squares represent oxygen vacancies. Almost all of the vacancies are at the O1 site. b) Thermodynamically‐stable ordered Sr_3_Fe_2_O_6_ (*δ* = 1.0). All of the vacancies are at the O1 site. c) The structural evolution of Sr_3_Fe_2_O_7−__*δ*_ without (w/o) Pd, and d) with (w/) Pd. e) Ideal vacancy‐diffusion pathways. f) Lattice constants of partially‐ and fully‐disordered (PD and FD) Sr_3_Fe_2_O_6_ models calculated from first principles. The geometrical means of the in‐plane lattice parameters are regarded as the *a*‐axis lengths of the disordered phases. g,h) The green‐filled circle shows the lattice parameters of the ordered Sr_3_Fe_2_O_6_, while the blue and red‐filled circles show calculated values for the PD and FD models, respectively. Representative structures are shown in **g** and **h**. The blue and red dashed lines are the averages of the PD and FD models, respectively.

The reaction from Sr_3_Fe_2_O_7−__*δ*_ (*δ* ∼ 0.4) to Sr_3_Fe_2_O_6_ (*δ* = 1.0) should involve oxygen deintercalation at the surface and oxygen diffusion in the bulk. Thus, oxygen vacancies must be randomly distributed at both O1 and O2 sites during bulk diffusion since the favorable oxygen conduction pathway in Sr_3_Fe_2_O_7−__*δ*_ is ‐O1‐O2‐O1‐O2 in the perovskite layer (**Figure** **4**e).^[^ ^15^ ^]^ However, most of the vacancies are at the O1 site in the thermodynamically stable structures (Figure 4a,b).^[^ ^12^ ^]^ Therefore, it is expected that a nonequilibrium dynamically‐disordered phase can emerge before the oxygen vacancies are rearranged to make thermodynamically stable structures.

The experimental observation showed the pristine Sr_3_Fe_2_O_7−__*δ*_ reacts slowly, with a continuous change in structure, while Pd/Sr_3_Fe_2_O_7−__*δ*_ reacts quickly, with an obvious jump in the lattice parameters. We propose that this change in the reaction pathway is governed by the rate‐determining step of each reaction. In the reduction of pristine Sr_3_Fe_2_O_7−__*δ*_, the surface reaction is slower than bulk diffusion because of slow H_2_ dissociation, which was previously determined by temperature‐programmed reactions.^[^ ^8^ ^]^ As a result, the structural relaxation, i.e. rearrangement of oxygen vacancies, is faster than the vacancy supply. This allows a continuous relaxation through thermodynamically‐stable phases, resulting in a continuous change in the lattice parameters (Figure 1e). In contrast, in the case of Pd/Sr_3_Fe_2_O_7−__*δ*_, Pd metal nanoparticles on Sr_3_Fe_2_O_7−__*δ*_ dramatically accelerated H_2_ adsorption and dissociation (spillover effect), which facilitated the reaction between lattice oxygen and H_2_.^[^ ^13^, ^14^ ^]^ As a result, the surface reaction rate exceeds the bulk diffusion rate in the reduction of Pd/Sr_3_Fe_2_O_7−__*δ*_ by surface modification. This change of the rate‐determining step is supported by previous density functional theory (DFT) calculations: It has been shown that the H_2_ adsorption and dissociation on Pd metal surface is energetically very favorable.^[^ ^14^ ^]^ The activation energy for the path, in which the hydrogen dissociated on Pd metal surface transfers to the catalyst support, is only 0.58 eV.^[^ ^14^ ^]^ This value is much lower than that (1.09 eV) for the diffusion of lattice oxygen in Sr_3_Fe_2_O_7−__*δ*_ which is obtained from the same calculation method.^[^ ^15^ ^]^ As the result of the change of the rate‐determining step, the vacancy supply becomes faster than the structural relaxation so that the *δ* value reaches a maximum (*δ* = 1.0) before the structure fully relaxes. This allows us to observe the first‐order transition from dynamically‐disordered Sr_3_Fe_2_O_6_ to ordered Sr_3_Fe_2_O_6_ (Figure 4d).

Now let us explain the reaction pathway for the pristine Sr_3_Fe_2_O_7−__*δ*_ without the nonequilibrium phase (Figure 4c). As seen in Figure 1e, the time dependence of the lattice parameters obtained by Le Bail analysis reveals that the *a*‐axis length monotonically increases. In contrast, the *c*‐axis length continuously increases until 3 s after the H_2_ injection and then gradually decreases. The nonmonotonic evolution of the *c*‐axis can be rationalized by considering two competing effects: One is expansion by decreasing the valence of Fe ions; the other is reduction of the Fe‐Fe distance along the *c*‐axis by deintercalation of oxide ions at the O1 site to form face‐to‐face FeO_5_ pyramids. A schematic image of the structural evolution is shown in Figure 4c. In the initial stage of the reduction, the former effect is dominant because the residual oxide ions at the O1 site work as pillars to maintain the long Fe‐Fe distance. As the *δ* value increases, the latter effect becomes dominant because the “pillar” oxide ions are deintercalated. Although the reaction rate decreases by decreasing the reaction temperature, the features of the lattice evolutions are similar at all temperatures between 673 K and 773 K (Figure S11, Supporting Information).

In Pd/Sr_3_Fe_2_O_7−__*δ*_, both the *a‐* and *c*‐axis lengths continuously increase before the discrete transition (Figure 1g), corresponding to a volume expansion (Figure 1h) due to the decrease in Fe valence. During the transition, the *a‐*axis jumps and the *c*‐axis drops (Figure 1g). The drop of the *c*‐axis corresponds to the vacancy ordering since oxide ions at the O1 site in the disordered phase must work as pillars to maintain the long Fe–Fe distance as discussed above. The small difference in volume before and after the transition (Figure 1g) indicates that the phase just before the transition has the same chemical formula of Sr_3_Fe_2_O_6_. Namely, the rearrangement of oxygen vacancies occurs without a distinct change to the chemical formula at the transition. Thus, the nonequilibrium phase can be assigned as dynamically‐disordered Sr_3_Fe_2_O_6_ (or Sr_3_Fe_2_O_7−__*δ*_ (*δ* ∼ 1)), and the first‐order transition can be rationalized as being an order‐disorder transition in Sr_3_Fe_2_O_6_ (Figure 4d).

To capture the characteristics of the dynamically‐disordered phase at the atomic scale, we carried out a series of DFT calculations. As reported in Ref. 15, the possible oxygen conduction pathway is ‐O1‐O2‐O1‐O2 (Figure 4e). Taking this scenario into account, the oxygen vacancies in the disordered structure would be preferentially located at the O1 and O2 sites. Another possibility is that the oxygen vacancies exist at all the oxygen sites. We call these structures partially disordered (PD) and fully disordered (FD), respectively. We constructed ten models of Sr_3_Fe_2_O_6_, with a 4×4×1 supercell, for each, and calculated their lattice parameters.

As can be seen in Figure 4f, all the calculated disordered models of Sr_3_Fe_2_O_6_ have a longer *c‐*axis than that of the ordered model built from the experimental data (Figure 1b). The reason for this is that the partially occupied O1 atoms work as pillars to maintain the long Fe‐Fe distance along the *c*‐axis, as discussed above. Remarkably, the *a*‐axis lengths in the FD models tend to elongate compared with the ordered Sr_3_Fe_2_O_6_ but to decrease in the PD models. Since the trend in the PD models are qualitatively consistent with the experiments (Figure 1g), our DFT calculations imply that O1‐O2 site disorder (PD) is more realistic than full disorder (FD). We should, however, note that the elongation of the *c*‐axis in the PD model is larger (+2.9%) than experimentally observed (<0.8%, Figure 1g). The reason is not clear yet, but it is probably related to the time resolution of the experiments or the too‐small unit cell of the models, where the oxygen vacancies are periodically located at short intervals even in the disordered models.

## 3 Conclusion

We showed the emergence of nonequilibrium intermediate phases during fast oxygen deintercalation reaction of Pd‐loaded Sr_3_Fe_2_O_7−__*δ*_. The characterization of such a short‐lifetime intermediate phase, which requires advanced techniques, is an essential step to understand the mechanism of the fast reactions. The current compound is promising for a high‐performance oxygen storage material in catalytic systems for the purification of automotive exhaust gas, and Fe‐site‐substitution further improves the oxygen storage performance.^[^ ^7^, ^16^ ^]^ Therefore, application of the time‐resolved synchrotron X‐ray techniques to the substituted system to compare the difference in the reaction pathways will provide important information to optimize the oxygen storage performance.

Furthermore, we have demonstrated that the reaction process can indeed be manipulated by using a surface treatment to control the rate‐determining step in crystalline material (Figure 4c,d). Regarding the current reactions, the final products are the same despite the different reaction pathways. However, the selectivity of the reaction pathways will provide an opportunity for rational design of compounds. For example, in order to design organic molecules, organic chemists have developed protecting groups that are used to control the reaction pathway by limiting the diffusion of reactant molecules to the blocked sites. As demonstrated in this study, a similar reaction control through the surface treatment may be possible in crystalline phases. In fact, heavily hydrogen doped‐MoO_3_ and WO_3_ were reported by a low‐temperature reduction of metal‐loaded MoO_3_ and WO_3_ via a facile H‐spillover approach.^[^ ^17^ ^]^ Thus, metastable phases, which cannot be reached from a pristine sample, will be synthesized by the reduction of surface‐modified sample with appropriate temperature control.

## 4 Experimental Section

### Preparation of Powder Samples

Sr_3_Fe_2_O_7−__*δ*_ samples were prepared by a Pechini method according to the previous reports.^[^ ^8^, ^11^ ^]^ The typical procedure was as follows. 333 mmol of citric acid (98.0%, Wako Pure Chemicals) was dissolved in 180 ml of water at 353 K. Then, strontium carbonate (20 mmol, 99.9%, Wako Pure Chemicals) and iron nitrate nonahydrate (13.3 mmol, 99.9%, Wako Pure Chemicals) were added to obtain the solution containing the metal oxide complexes. To this solution, 333 mmol of ethylene glycol (99.5%, Wako Pure Chemical Industries) was added, and the solution was stirred at 403 K to obtain a gelatinous solution. After the gel was heated with a mantle heater at 623 K, the obtained powder was calcined at 1273 K. Pd‐loaded sample was synthesized by an impregnation method. Pd acetate (99.9%, Sigma‐Aldrich) was used as the Pd source material. Pd acetate was dissolved in acetone (99.0%, Wako Pure Chemicals) at room temperature. Then, Sr_3_Fe_2_O_7−__*δ*_ was added into the solution and the solution was evaporated at 353 K. After drying the solution containing the Pd precursor and Sr_3_Fe_2_O_7−__*δ*_, the obtained powder was calcined at 1073 K. The loading amount of Pd species was 1.0 wt% on a metal basis.

### Characterization of Powder Samples

To analyze the sample quality, we conducted XRD measurements using a D8 ADVANCE diffractometer (Bruker AXS) with Cu *K*_*α*_ radiation. The oxygen storage and release properties were examined using isothermal thermogravimetry (STA 2500 Regulus, NETZSCH) under H_2_‐O_2_ cycles. The sample (100 mg) was heated to 773 K in 2% O_2_/Ar. At this temperature, the inlet gas was switched to 2% H_2_/Ar, 2% O_2_/Ar, or 100% O_2_. The total gas flow rate was 100 mL min^−1^.

### Time‐Resolved Synchrotron XRD Measurements

Time‐resolved synchrotron XRD measurements were performed using a powder diffractometer equipped with MYTHEN detectors at the beamline BL02B2 (JASRI, SPring‐8). Incident beams from a bending magnet were monochromatized to *λ* = 0.775 Å. The data were collected with a flat panel detector. Finely ground powder samples were sieved through a 32‐µm mesh sieve and were packed into borosilicate or quartz capillaries with an diameter of 0.5 mm. The sample temperature was controlled by hot N_2_ gas flow devices, and the sample atmosphere was controlled with a remote gas‐ and vapor‐pressure control (RGVPC) system.^[^ ^4^ ^]^ First, the sample was heated to 973 K under H_2_ atmosphere, and pretreated by three cycles of O_2_ and H_2_ gas conditions for each 5 min. Then, the sample temperature was set to 773 K under H_2_, and the sample was oxidized by injection of O_2_ gas. After the evacuation of the sample space, the data collection was started 3 s before the H_2_ injection (≈0.5 atm). The interval between the diffraction measurements on Sr_3_Fe_2_O_7−__*δ*_ (or Pd/Sr_3_Fe_2_O_7−__*δ*_) was 200 (or 100) ms. Several cycles for the redox reaction were carried out to confirm the reproducibility. The collected profiles were analyzed by the Le Bail method using JANA 2006,^[^ ^18^ ^]^ and TOPAS software.

### Simultaneous Time‐Resolved Quick X‐Ray Absorption Fine Structure QXAFS – XRD Measurements

Simultaneous time‐resolved synchrotron measurements were performed at the beamline BL36XU (JASRI, SPring‐8). The instrumental setup was reported elsewhere.^[^ ^19^ ^]^ Finely ground powder samples of Pd/Sr_3_Fe_2_O_7‐__*δ*_ were sieved through a 32‐µm mesh sieve and packed into quartz capillaries with an diameter of 0.5 mm. The sample atmosphere was controlled with RGVPC system.^[^ ^4^ ^]^ Incident beams from an undulator were monochromatized to *λ* = 1.746456 Å during XRD measurements. First, the sample was heated to 923 K under an H_2_ atmosphere, and pretreated by three cycles of O_2_ and H_2_ gas conditions for each 10 min. Then, the sample temperature was set to 773 K under H_2_, and the sample was oxidized by injection of O_2_ gas. After the evacuation of the sample space, the data collection was started 3 s before the H_2_ injection (≈1 atm). The interval between the diffraction measurements was 100 ms (20 ms and 80 ms. exposure times for XRD and QXAFS, respectively).

### Time‐Resolved In Situ Energy‐Dispersive XAFS (DXAFS) Measurements

Time‐resolved in‐situ DXAFS at Sr K‐edge was measured at the beamline BL28B2 of SPring‐8. The sample (0.03 g) was pretreated under pure H_2_ and O_2_ at 823 K. The in situ cell was evacuated, and then pure H_2_ was injected into the oxidized sample. The XAFS spectra were recorded every 70 ms. at 823 K. For details of the experimental conditions, please refer to the previous paper.^[^ ^8^ ^]^

### First‐Principles Calculations

The first‐principles calculations were performed using the projector augmented‐wave (PAW) method^[^ ^20^ ^]^ implemented in VASP.^[^ ^21^ ^]^ PAW datasets with radial cutoffs of 1.32, 1.22, and 0.80 Å were employed for Sr, Fe, and O, respectively. Sr 4*s*, 4*p*, and 5*s*, Fe 3*d* and 4*s*, and O 2*s* and 2*p* orbitals were considered as valence electrons. The Perdew–Burke–Ernzerhof generalized gradient approximation^[^ ^22^ ^]^ with the Hubbard *U* correction^[^ ^23^ ^]^ was adopted, where *U*_eff_ for the Fe 3*d* orbitals is set to 4.0 eV.^[^ ^15^ ^]^ The initial magnetic configurations were set to the G‐type anti‐ferromagnetic ordering.^[^ ^15^ ^]^ The 4×4×1 supercells with the Γ‐only *k*‐point sampling were used for modeling the ordered and disordered Sr_3_Fe_2_O_6_. The plane‐wave cutoff energy was set to 600 eV. The structure optimizations were continued until the residual forces on atoms and stresses were reduced to less than 0.03 eV Å^−1^ and 0.04 GPa. The settings for the VASP code were supported by the vise code (version 0.6.1).^[^ ^24^ ^]^

## Conflict of Interest

The authors declare no conflict of interest.

## Author Contributions

T.Y. and S.K. contributed equally to this work. T.Y., S.K., and S.H. designed the research. T.Y., T.K., A.S., K.B., T.T., M.A., and S.H. synthesized and characterized the samples. T.Y., S.K., T.K., A.S. K.B., and S.H. performed synchrotron XRD measurements. T.Y., T.K., K.B., T.O., T.N., K.H., K.N., T.U., S.Y., and S.H. performed QXAFS‐XRD measurements. K.B., K.K., and S.H. analyzed DXAFS measurements. N.T., Y.K., and F.O. carried out our DFT calculations. T.Y. and S.H. wrote the manuscript, with comments from all the authors.

## Supporting information

Supporting Information

## Data Availability

The data that support the findings of this study are available from the corresponding author upon reasonable request.
