# Supplementary material for: Emergence of Dynamically‐Disordered Phases During Fast Oxygen Deintercalation Reaction of Layered Perovskite
Source: Adv Sci (Weinh). 2023 Apr 25;10(19):2301876. doi: 10.1002/advs.202301876 (PMC10323665; doi:10.1002/advs.202301876)
Supplement: Supplementary file 1 — Supporting Information [file ADVS-10-2301876-s001.pdf]

ADVANCED SCIENCE
Open Access

Supporting Information

for *Adv. Sci.*, DOI 10.1002/advs.202301876

Emergence of Dynamically-Disordered Phases During Fast Oxygen Deintercalation Reaction of Layered Perovskite

Takafumi Yamamoto, Shogo Kawaguchi, Taiki Kosuge, Akira Sugai, Naoki Tsunoda, Yu Kumagai, Kosuke Beppu, Takuya Ohmi, Teppei Nagase, Kotaro Higashi, Kazuo Kato, Kiyofumi Nitta, Tomoya Uruga, Seiji Yamazoe, Fumiyasu Oba, Tsunehiro Tanaka, Masaki Azuma and Saburo Hosokawa**

Supporting Information

Emergence of dynamically-disordered phases during fast oxygen deintercalation reaction of layered perovskite

T. Yamamoto *et al*.

Contents: **Figure S1-S11**

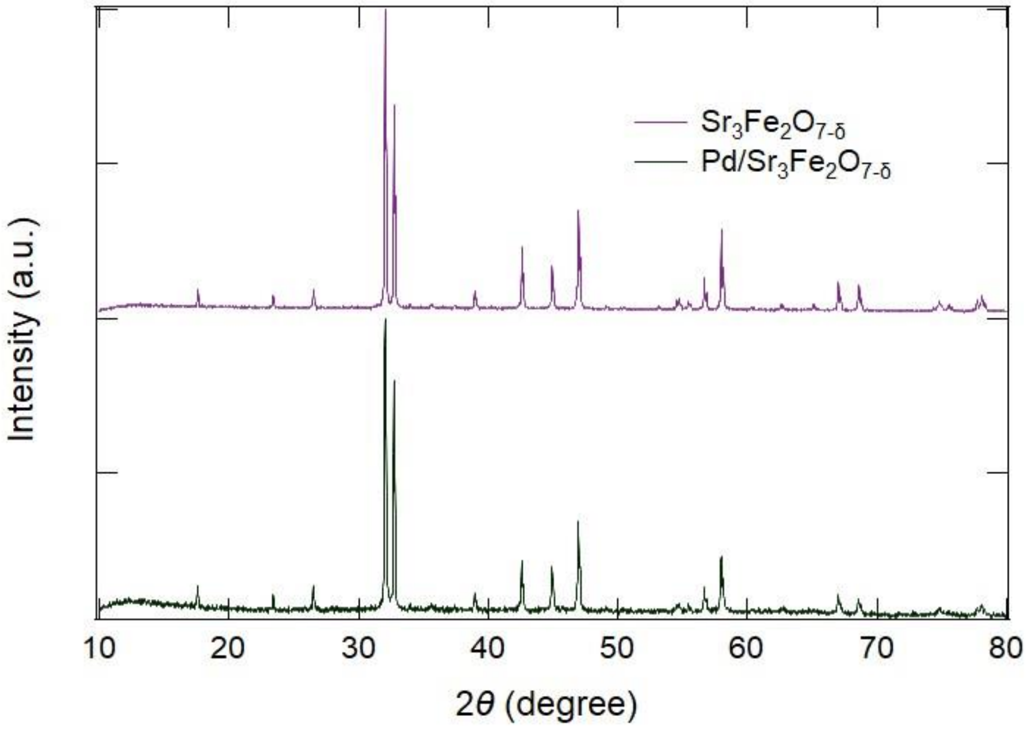

Fig. S1. Laboratory XRD patterns for as-prepared $Sr_3Fe_2O_{7-\delta}$ and Pd/$Sr_3Fe_2O_{7-\delta}$ at room temperature.

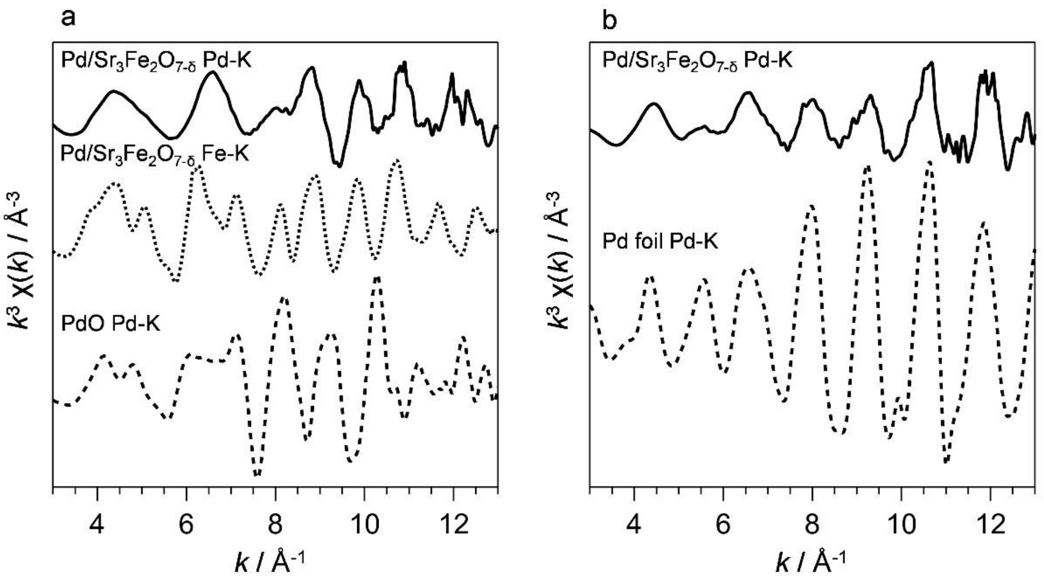

Fig. S2. Pd and Fe K-edge EXAFS oscillations obtained in a static XAFS measurements of Pd/$Sr_3Fe_2O_{7-\delta}$ before (a) and after reduction under H_2 flow at 773 K (b).

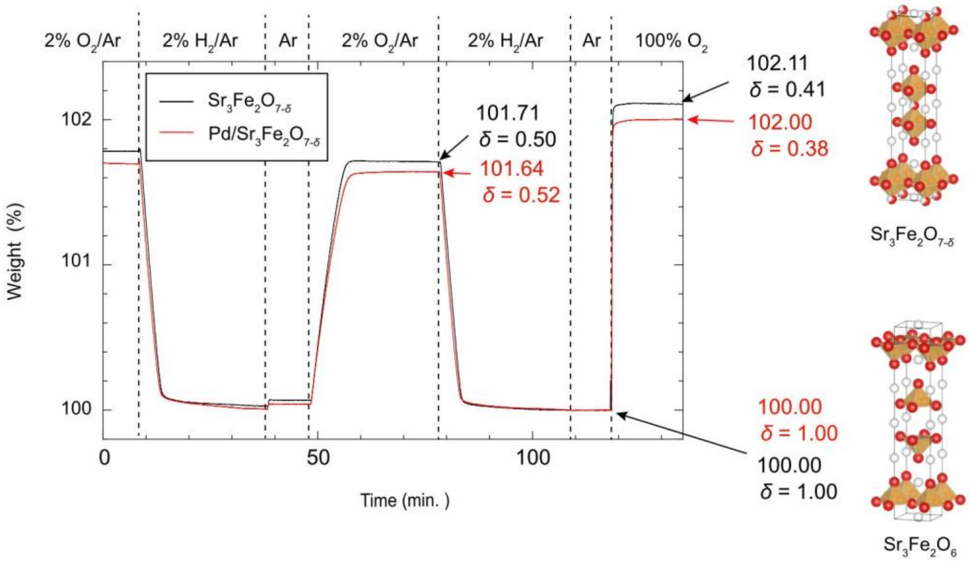

Fig. S3. Thermogravimetric analyses for $Sr_3Fe_2O_{7-\delta}$ and Pd/$Sr_3Fe_2O_{7-\delta}$ at 773 K. $Sr_3Fe_2O_{7-\delta}$ (100.75 mg) and Pd/$Sr_3Fe_2O_{7-\delta}$ (108.355 mg) were heated to 773K under 2 % O_2/Ar. The δ for $Sr_3Fe_2O_{7-\delta}$ and Pd/$Sr_3Fe_2O_{7-\delta}$) under 100% O_2 are calculated as $\delta = 0.41$ and 0.38, respectively.

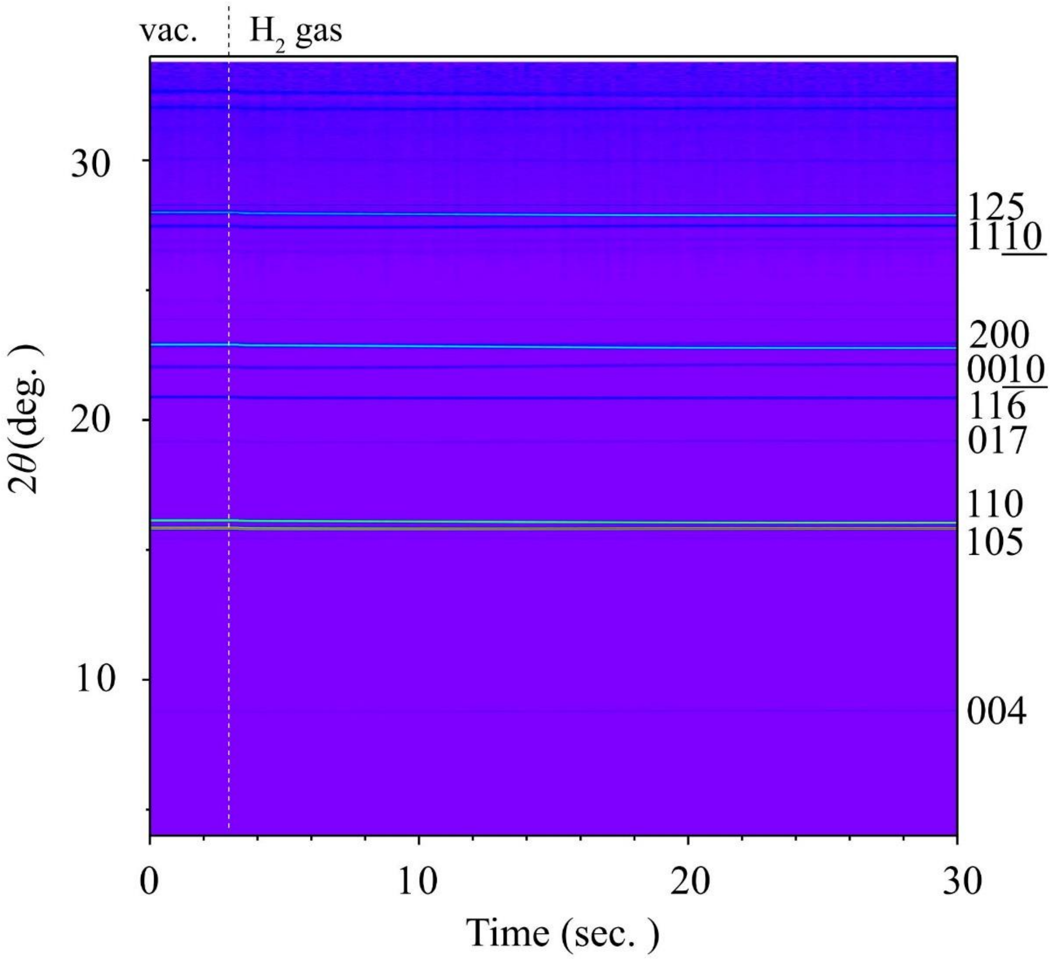

Fig. S4. Full angle data of time-resolved XRD patterns of reduction reaction for $Sr_3Fe_2O_{7-\delta}$ at 773 K. Data around the main peaks are shown in Figure 1c in the main text.

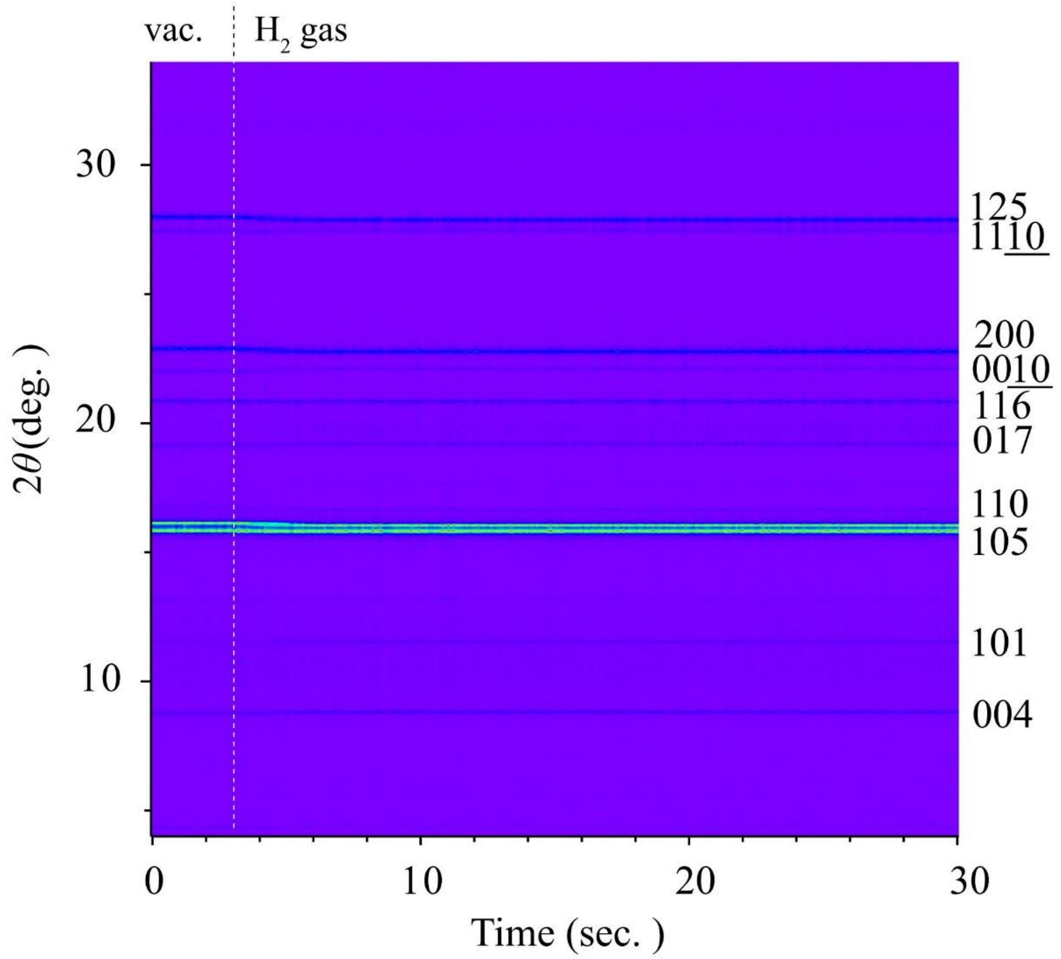

Fig. S5. Full angle data of time-resolved XRD patterns of reduction reaction for Pd/$Sr_3Fe_2O_{7-\delta}$ at 773 K. Data around the main peaks are shown in Figure 1d in the main text.

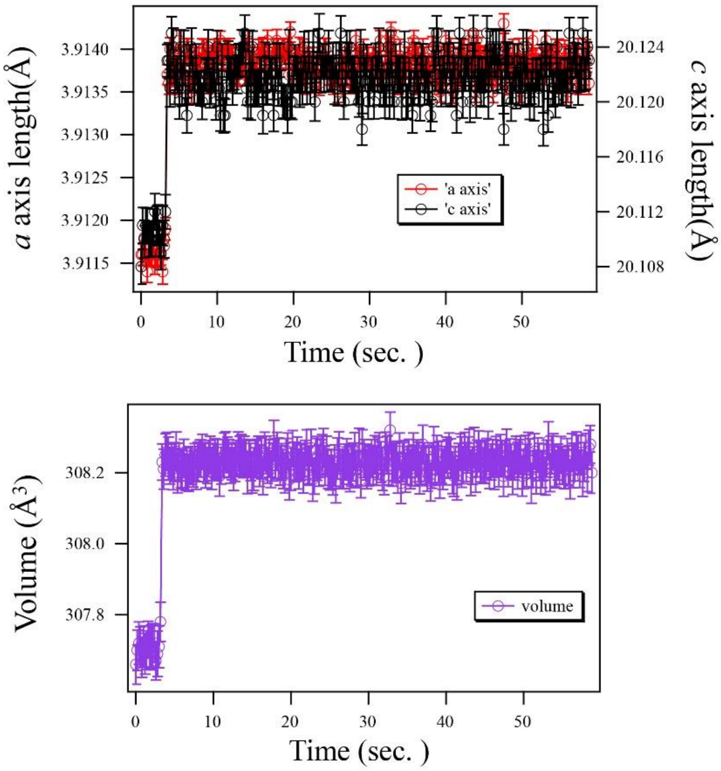

Fig. S6. Time profiles of lattice parameters obtained from time-resolved XRD measurements for $Sr_3Fe_2O_6$ from vacuum to Ar gas at 723 K. The initial condition was vacuum, and then the Ar gas (~ 50 kPa) was injected the 3 second after the starting the measurements. All the parameters increase immediately after the injection. The increase of temperature by the injection is estimated to approximately 60 K, which was calculated from volume expansion of $Sr_3Fe_2O_6$ from 307.71 Å^3 to 308.23 Å^3(volume expansion coefficient of 8.4×10^{-3} Å^3/K is estimated from data taken at 673 K, 723K and 773K).

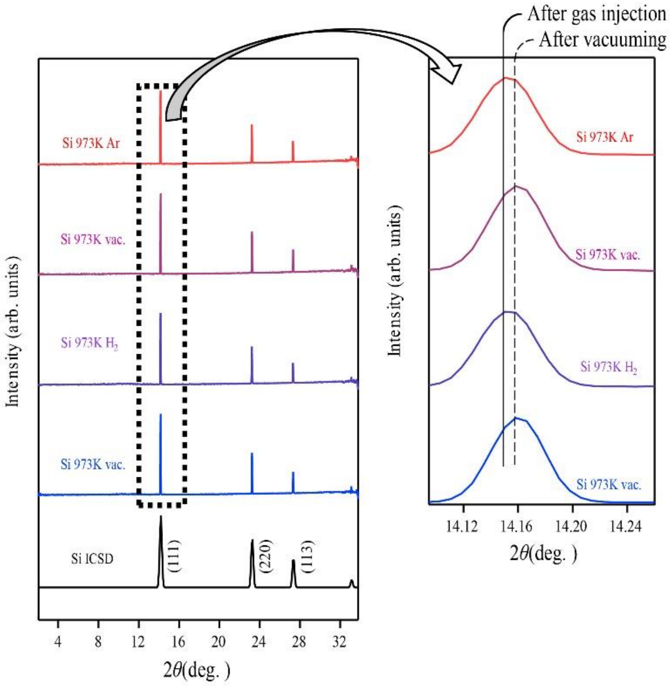

Fig. S7. XRD patterns of Si from vacuum under various conditions at 973 K. The peak positions under vacuum are higher than those of H_2 and Ar, suggesting the sample temperature is lower under vacuum condition due to adiabatic expansion. The decrease of the temperature under vacuum is estimated to be approximately 150 K from the lattice parameters.

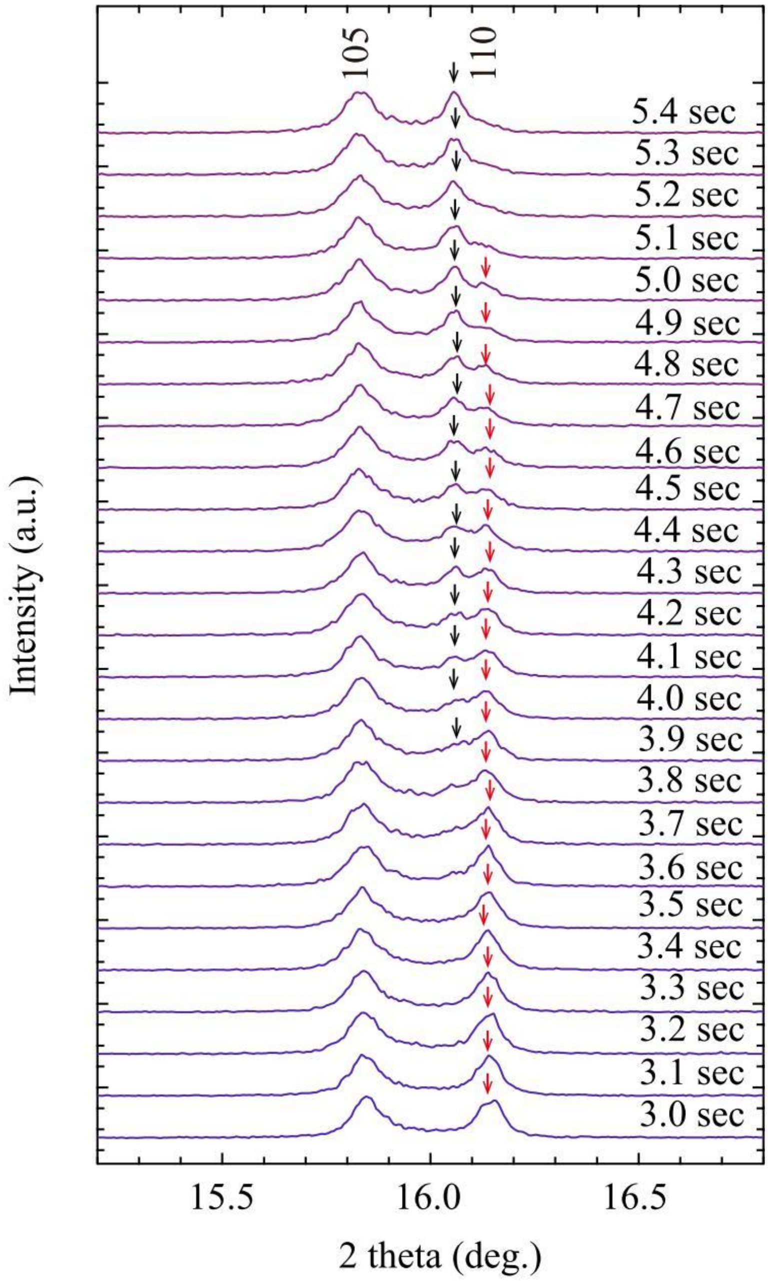

Fig. S8. 1D profiles of Time-resolved XRD data of BL02B2 for reduction reaction of Pd/$Sr_3Fe_2O_{7-\delta}$ at 773K. Two phases coexist between 4 – 5 seconds.

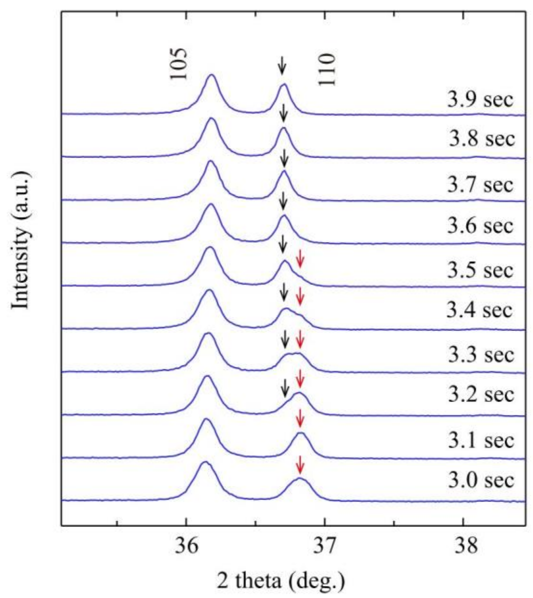

Fig. S9. 1D profiles of Time-resolved XRD data of BL36XU for reduction reaction of Pd/$Sr_3Fe_2O_{7-\delta}$ at 773K. Two phases coexist between 3.2 - 3.5 seconds.

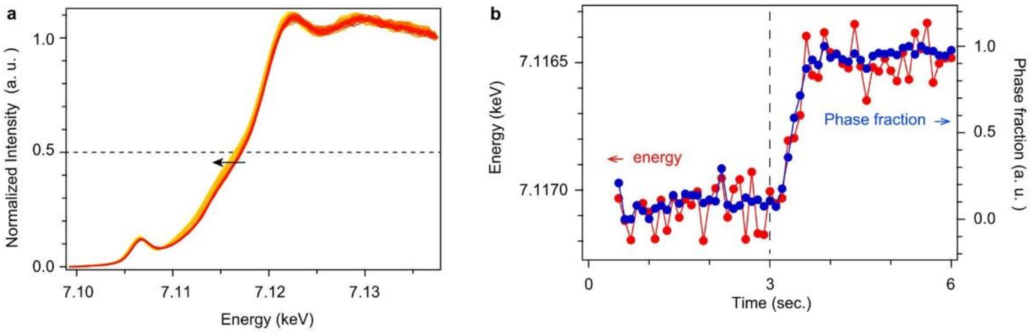

Fig. S10. Energy shift of normalized QXAFS spectra. 1D profiles of normalized QXAFS spectra (0 sec – 6.0 sec) (a) and Energy shift from normalized QXAFS spectra at half of the intensity for reduction reaction of Pd/$Sr_3Fe_2O_{7-\delta}$. Phase fractions in (b) are equal to those in Figure 2d in the main text.

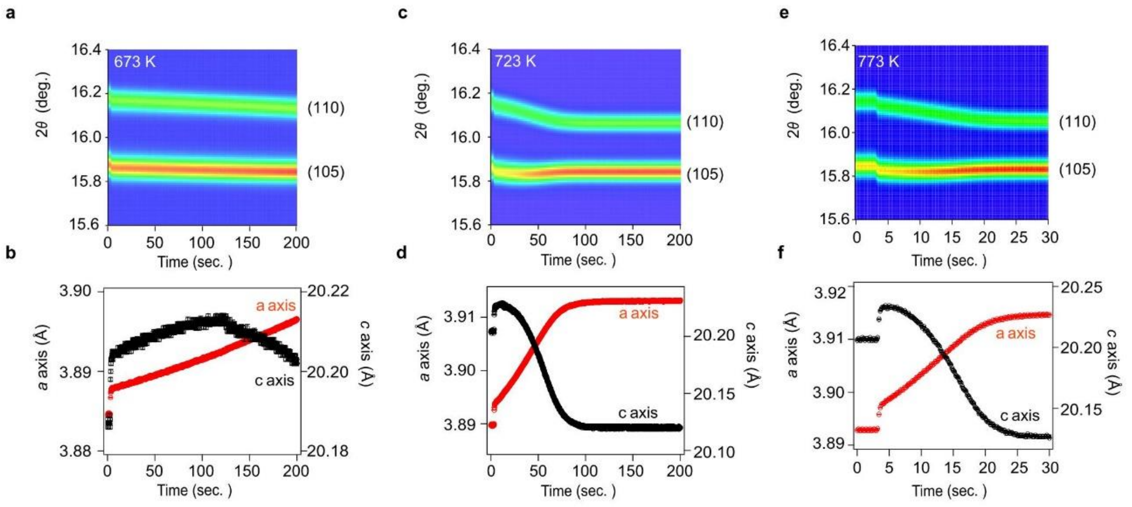

Fig. S11. Time-resolved XRD measurements of reduction reaction for $Sr_3Fe_2O_{7-\delta}$ under various temperatures. Time-resolved XRD patterns at 673 K (a), 723 K (c), and 773K (e). Time profiles of the lattice parameters at 673 K (b), 723 K (d), and 773K (f). Note that the time range of horizontal axis in (e) and (f) is 15 times shorter than those of (a) to (d).
